# PARP inhibitors as radiosensitizers: a comprehensive review of preclinical evidence and clinical applications

**DOI:** 10.3389/fonc.2025.1702121

**Published:** 2025-11-24

**Authors:** Xiaodong Luo, Shuo Gao, Kaiyang Sun, Zhiqiang Dong, Shude Yang

**Affiliations:** 1 Department of Neurosurgery, Lanzhou University Second Hospital, Lanzhou, China; 2 Lanzhou University, Lanzhou, China

**Keywords:** PARP1, radiotherapy, DNA damage repair, PARP inhibitors, radiosensitization, synthetic lethality, tumor microenvironment, precision therapy

## Abstract

Poly (ADP ribose) polymerase 1 (PARP1) plays a central role in the response of DNA damage induced by tumor radiotherapy. This paper systematically reviewed the structural and functional characteristics of PARP1 and its molecular mechanism in DNA damage repair, and focused on the preclinical evidence and clinical transformation research progress of PARP inhibitor (parpi) as a radiosensitizer. PARP1 affects the effect of radiotherapy by recognizing DNA breakage, catalyzing par modification and regulating repair pathway, while parpi significantly enhances radiosensitivity by inhibiting DNA repair, inducing synthetic lethal effect and regulating immune microenvironment. Although preclinical studies have shown good prospects in a variety of solid tumors, clinical transformation still faces challenges such as heterogeneity of efficacy and drug resistance mechanism. Future research should focus on precise treatment strategies, joint scheme optimization and drug resistance mechanism exploration, so as to promote the wide application of parpi in radiotherapy. This article presents a narrative review of the preclinical and clinical evidence supporting the use of PARP inhibitors as radiosensitizers.

## 1 Introduction

Radiotherapy is a commonly used cancer treatment that employs high-energy ionizing radiation (such as X-rays and γ-rays) to directly or indirectly induce DNA damage (1). Direct effects involve radiation disrupting DNA structure, leading to single-strand breaks (SSBs) and double-strand breaks (DSBs), among other lesions (2, 3). Indirect effects occur through the activation of water molecules, generating free radicals that cause oxidative damage (4), resulting in cell cycle arrest or death. These damages trigger the ATM/ATR kinase cascade, activating a multi-pathway repair network including base excision repair (BER), homologous recombination (HR), and non-homologous end joining (NHEJ) (5). The efficacy of radiotherapy is closely related to DNA damage repair capacity, providing a rationale for intervention strategies aimed at enhancing radiosensitivity.

Poly(ADP-ribose) polymerase 1 (PARP1) plays a key regulatory role in the DNA damage response (DDR), with its function finely modulated by different structural domains (6). During radiotherapy, PARP1 participates in DNA damage repair primarily through three classical mechanisms: first, it recognizes and binds to DNA single- or double-strand breaks via auto-PARylation (7); second, it forms long, highly branched poly(ADP-ribose) (PAR) polymer chains that amplify DNA damage signals (8); and third, it activates XRCC1-mediated base excision repair (BER) to repair single-strand breaks (SSBs) (9). The activity level of PARP1 significantly influences radiotherapy outcomes: moderate activation aids DNA repair and maintains cellular homeostasis, whereas overactivation induced by high-dose radiation leads to severe NAD^+^/ATP depletion, causing energy metabolism collapse and a specific form of programmed necrotic cell death known as parthanatos [10, 11]. The critical role of PARP1 in radiotherapy response lies in its balanced regulation of repair efficiency and cell fate decisions. This dynamic regulatory mechanism is closely associated with radioresistance and toxic responses.

This narrative review comprehensively summarizes the central role of PARP1 in DNA damage repair and critically examines its promise as a target for radiosensitization in cancer radiotherapy. By synthesizing evidence from molecular mechanisms to clinical applications, we seek to provide a foundational framework and inform the rational development of next-generation radiosensitization strategies.

**Figure 1:**
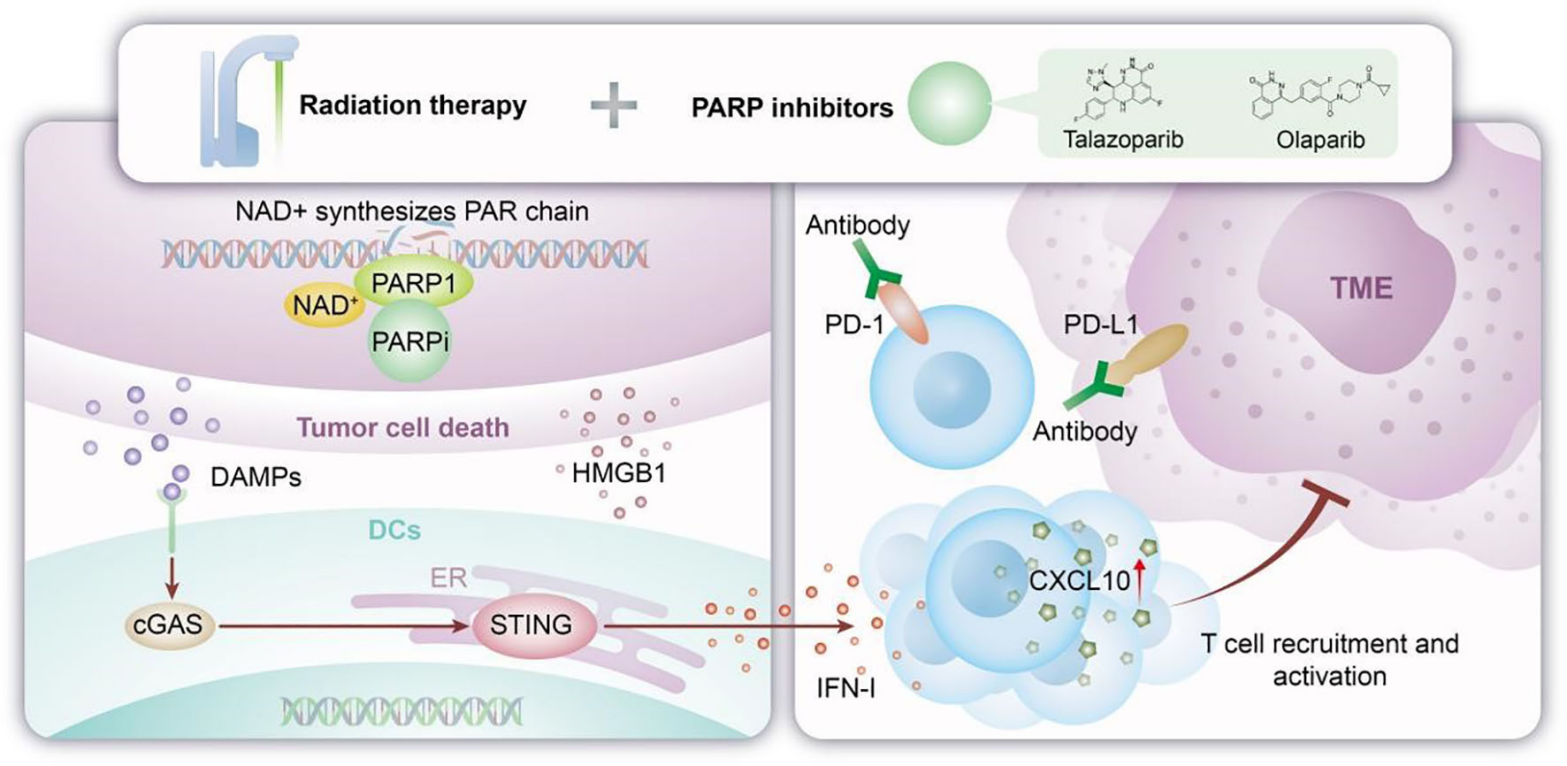
Mechanism of PARP inhibitor combined with radiotherapy.

Compared to existing reviews that often focus broadly on the DNA damage response or the clinical development of PARP inhibitors in monotherapy, this narrative review provides a dedicated and updated synthesis of the role of PARP inhibitors specifically as radiosensitizers. We place a particular emphasis on the translational journey—from the structural basis of PARP1 and its mechanistic interplay with radiation to the latest preclinical evidence and the practical challenges of clinical integration. A unique contribution of this work is its critical examination of the “double-edged sword” of normal tissue toxicity and its in-depth discussion on biomarker-driven patient stratification beyond the established HRD and BRCA status, incorporating emerging candidates like SLFN11, LP52, and functional PARP1-EJ assays. Finally, we consolidate these insights into a forward-looking roadmap designed to bridge the gap between preclinical promise and clinical reality, outlining prioritized research directions and actionable strategies for the field (**Figure 1**).

## 2 Methods

### 2.1 Review design and literature search strategy

This work is a narrative review designed to provide a comprehensive and critical overview of the role of PARP inhibitors as radiosensitizers in cancer therapy. To ensure a broad and representative coverage of the existing literature, a targeted literature search was conducted in the electronic databases PubMed and Web of Science. The search focused on articles published until July 2025.

The search strategy employed a combination of key terms related to: (1) “PARP inhibitor” OR “PARP1”; (2) “radiosensiti*” OR “radiation” OR “radiotherapy”; and (3) specific tumor types (e.g., “non-small cell lung cancer”, “triple-negative breast cancer”). The specific search strings are provided in **Supplementary Material S1**.

Given the narrative nature of this review, the study selection process was iterative and aimed at identifying seminal, high-impact, and illustrative studies. The inclusion criteria were: (1) original research articles and clinical trials; (2) studies investigating the combination of PARP inhibitors with ionizing radiation; (3) studies reporting on mechanistic insights, efficacy, or toxicity outcomes. Exclusion criteria included: (1) conference abstracts without full-text papers; (2) studies not directly relevant to the core topic.

The literature search and selection were performed by the authors. While a formal, multi-stage screening process (e.g., title/abstract followed by full-text) was not employed as in a systematic review, the final selection of cited literature was based on a consensus among the authors to best represent the current state of the field, from foundational mechanistic studies to the latest clinical trials. A PRISMA flow diagram is not applicable for this narrative review design.

## 3 Structural and functional characteristics of PARP1

The human PARP1 gene is located on chromosome 1q41-42, spans approximately 43 kb, and contains 23 exons (11, 12). Similar to other mammalian PARP1 promoters, the human promoter region lacks typical regulatory elements such as TATA or CAAT boxes (13, 14). The PARP1 transcript is 3042 nucleotides in length and contains an open reading frame encoding a 113 kDa protein composed of 1014 amino acids (15). Previous studies have identified a PARP protein family consisting of at least 17 members (16). Although these members share sequence homology with PARP1 in the catalytic domain, research has primarily focused on PARP1. As the most abundant and well-characterized isoform, PARP1 functions as a DNA damage nick sensor involved in DNA repair and the maintenance of genomic integrity (17).

### 3.1 Structural features of PARP1

The molecular structure of PARP1 consists of multiple functionally defined domains arranged linearly. Its N-terminal region contains a DNA-binding domain (DBD) composed of two highly conserved zinc finger motifs, F1 and F2, which cooperatively recognize and bind to DNA single- or double-strand break sites (18). This is followed by a nuclear localization signal (NLS) that mediates the post-translational transport of PARP1 into the nucleus. Subsequent studies identified an adjacent regulatory region containing a third zinc finger structure, F3, which shares no significant homology with F1 and F2 and does not directly participate in DNA binding but plays a critical role in the conformational activation of PARP1 and the initiation of its catalytic function (19).

The central region of PARP1 contains a BRCT domain (Breast Cancer Susceptibility Gene 1 C-Terminal domain), which serves as a protein-protein interaction platform involved in recruiting other DNA damage response factors (20). Additionally, the WGR domain (named after the conserved Trp-Gly-Arg residues initially identified within it) is located between the BRCT and catalytic domains and is believed to act as a bridge in sensing DNA damage and mediating structural rearrangements. The C-terminal region houses the catalytic domain (CAT), which contains the NAD^+^ binding site and is responsible for catalyzing the synthesis of poly(ADP-ribose) (PAR) chains, thereby playing a central role in DNA repair, maintenance of genomic stability, and regulation of cell death (21).

**Figure 2:**
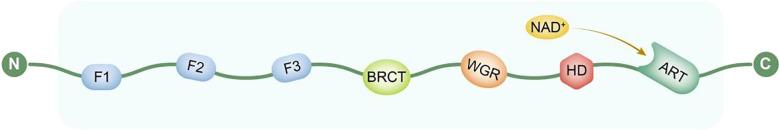
Structural features of PARP1.

These domains work in concert to enable PARP1 to efficiently sense DNA damage, recruit repair complexes, and regulate downstream signaling pathways (**Figure 2**).

### 3.2 Functional characteristics of PARP1

The poly(ADP-ribosyl)ation activity of PARP1 is known to significantly enhance in the presence of DNA damage, with the rate of PAR chain synthesis increasing several hundred-fold—a characteristic discovered as early as the 1980s (22). Consequently, research over the past few decades has primarily focused on elucidating the role of PARP1 in the DNA damage response (DDR) pathway, particularly its key regulatory functions in base excision repair (BER), single-strand break repair (SSBR), and the maintenance of genomic stability. Numerous studies indicate that PARP1 plays a crucial role in maintaining genetic integrity by recognizing DNA breaks, rapidly recruiting repair factors, and acting as a molecular scaffold to facilitate the assembly of repair complexes.

PARP1 targets damage sites through specific and dynamic binding modes, playing a pivotal role in DNA damage repair. Its association with chromatin is primarily mediated by its ability to directly recognize various DNA damage structures, a process that can synergize with nucleosome structures and interactions with chromatin-associated proteins, forming a multivalent, non-mutually exclusive binding mechanism. PARP1 efficiently recognizes and binds to multiple DNA damage-related conformations, including single- and double-strand breaks, DNA crosslinks, cruciform structures (such as Holliday junctions), and non-B DNA structures like negatively supercoiled regions (23). Upon binding to DNA, PARP1 undergoes a dramatic conformational change, activating it into a highly catalytically active state (approximately 500-fold increase) (21, 24). Additionally, studies have shown that the protein can also bind to certain specific sequences of double-stranded DNA, suggesting a potential preferential localization tendency in specific genomic regions (25). These properties enable PARP1 to rapidly locate break sites during the early stages of DNA damage, initiating downstream repair cascades, including the recruitment of repair factors, promotion of chromatin decompaction, and assembly of repair complexes as a molecular scaffold, thereby serving as a core sensor in pathways such as BER and SSBR.

Following DNA binding, PARP1 catalyzes the conversion of NAD^+^, utilizing the resulting ADP-ribose monomers to synthesize structurally complex, long, and branched poly(ADP-ribose) (PAR) polymers. These PAR chains are covalently attached to PARP1 itself (auto-PARylation) and to various nuclear proteins around the damage site (trans-PARylation), thereby amplifying the DNA damage signal (8). In subsequent repair cascades, PARP1 and the scaffold protein XRCC1 form a core regulatory axis: the PAR chains generated by PARP1 are specifically recognized by the BRCT-I domain of XRCC1, anchoring it to the damage site (26). The two form a negative feedback loop: XRCC1 inhibits excessive auto-PARylation of PARP1, preventing NAD^+^ depletion and protecting damaged ends, while simultaneously constructing a repair platform to recruit factors such as POLβ and LIG3α, efficiently coordinating BER (27). This mechanism is a core defense strategy against oxidative damage, and its dysregulation leads to abnormal PAR accumulation, repair failure, and cell death.This hyperactivation and the ensuing energy crisis represent one arm of the cytotoxic effects of PARP inhibition. The primary therapeutic window, however, is provided by the concept of synthetic lethality in HR-deficient tumors, such as those with BRCA mutations, where PARP inhibition leads to the accumulation of irreparable DNA damage (28).

## 4 Comparative profiles of clinically approved PARP inhibitors

Currently, several PARP inhibitors are widely used in clinical practice, primarily including Olaparib, Rucaparib, Niraparib, and Talazoparib. Although these drugs all belong to the PARP enzyme inhibitor class structurally, they exhibit significant differences in catalytic inhibitory activity, PARP-trapping capacity, pharmacokinetic properties, and clinical toxicity profiles (29).

PARP-trapping capacity is considered a primary mechanism of their cytotoxicity. Talazoparib is the most prominent in this aspect, with a trapping potency approximately 100-fold greater than that of Olaparib and Rucaparib, significantly exceeding other drugs (30). This also explains its notably lower clinical dosage (1 mg/day). Furthermore, differences in the half-lives of various PARP inhibitors influence their dosing regimens: Talazoparib and Niraparib have longer half-lives, allowing for once-daily administration, whereas Olaparib and Rucaparib require twice-daily dosing.

Regarding safety, common class-effects include hematological toxicity (such as anemia and thrombocytopenia) and gastrointestinal reactions. However, the specific toxicity profile and incidence rates vary among the drugs. For instance, Niraparib is associated with a higher risk of thrombocytopenia (31). In terms of use in special populations, Talazoparib is primarily excreted renally, requiring dose adjustment in patients with renal impairment; whereas Niraparib requires dose reduction in patients with hepatic impairment. Additionally, the blood-brain barrier penetration capacity differs among these drugs, which may influence their efficacy against brain metastases. The table below summarizes a comparison of these key parameters. A comprehensive comparison of these key pharmacological and clinical parameters is provided in **Table 1**.

**Table 1:** Comparative pharmacological profiles of clinically approved PARP inhibitors.

Parameter	Olaparib	Rucaparib	Niraparib	Talazoparib
PARP Trapping Potency	1 (Reference)	~1 (Similar to Olaparib)	Lower than Talazoparib	Very High (~100x Olaparib)
Catalytic Inhibition IC_50_ (nM)	6	21	60	4
Half-life (hours)	~15	~26	~36	~90
Dosing Regimen	Twice Daily	Twice Daily	Once Daily	Once Daily
Clinical Dose	300 mg, BID	600 mg, BID	200 or 300 mg, QD	1 mg, QD
BBB Penetration	Limited	Limited	Limited	Evidence of penetratio
Key Toxicity Profile	Anemia, Fatigue, Nausea	Fatigue, Elevated LFTs, Nausea	Thrombocytopenia, Anemia, Hypertension	Anemia, Neutropenia
Dose Adjustment in Hepatic Impairment	Not studied in severe impairment	Not studied in severe impairment	Reduce dose in moderate impairment	Not required
Dose Adjustment in Renal Impairment	Reduce dose in moderate impairment	Not required in mild-moderate impairment	Not required in mild-moderate impairment	Reduce dose in moderate & severe impairment
Primary Metabolic Pathway	CYP3A4/5	CYP2D6 (major), CYP1A2, CYP3A4	Carboxylesterases	Minimal hepatic metabolism, primarily renally excreted unchanged
Drug-Drug Interaction Potential	High (Avoid strong CYP3A inducers/inhibitors)	Moderate (Acts as a BCRP/CYP3A inhibitor)	Not fully studied	Low (Primarily with P-gp inhibitors)

Data on catalytic inhibition, PARP-trapping potency, pharmacokinetics, and dosing are synthesized from a comprehensive review of the literature by Zeng et al. (29), which consolidated findings from key preclinical and clinical studies. Toxicity profiles and dose adjustment recommendations are based on the same source, which references official prescribing information and clinical trial data.

## 5 Mechanisms of PARP1-targeted radiosensitization and preclinical evidence

Shall et al. first reported the cytotoxic effects of PARP-1 inhibitors, demonstrating that the rejoining of DNA strand breaks could be inhibited by 3-aminobenzamide (32). PARP inhibitors (PARPis) structurally mimic nicotinamide and primarily exert dual functions: catalytic inhibition of PARP1 (preventing its PARylation) and trapping PARP1 onto damaged DNA (33, 34). Although the trapping mechanism is not fully elucidated, two hypotheses exist: inhibition of auto-PARylation may prevent PARP1 dissociation from DNA, or PARPi binding to the catalytic site may induce allosteric effects that enhance its DNA affinity. Regardless of the mechanism, trapped PARP1 stalls DNA replication forks. In tumor cells with defects in homologous recombination (HR) genes such as BRCA1/2, cells are forced to employ error-prone repair pathways like NHEJ/Alt-EJ, ultimately leading to chromosomal fragmentation and cell death—a phenomenon known as synthetic lethality (28). Based on this robust theoretical mechanism of PARPi-induced synthetic lethality (particularly via trapping), researchers rapidly explored the potential of PARPis as radiosensitizers in preclinical models. Multiple studies using *in vitro* cell models and *in vivo* animal models have provided strong evidence that PARPis can significantly enhance tumor cell sensitivity to radiation.

In studies on non-small cell lung cancer (NSCLC), the combination of olaparib and radiotherapy demonstrated significant synergistic effects. The mechanism is mainly twofold: first, by upregulating γH2AX expression, it enhances radiation-induced DNA double-strand breaks, thereby increasing tumor cell damage (35); second, by increasing the Bax/Bcl-2 ratio and activating Caspase-3 expression, it promotes tumor cell apoptosis. Notably, under hypoxic conditions (1% O_2_), olaparib exhibited stronger radiosensitizing effects in the Calu-6 cell line, which is closely related to homologous recombination repair (HR) deficiency—RAD51 downregulation under hypoxia triggers synthetic lethality (36). Furthermore, studies revealed that olaparib can enhance radiotherapy efficacy by improving tumor vascular perfusion (37). Recently developed olaparib nanoparticles (Ola-NPs) further increased the sensitization enhancement ratio (SER) to 3.81, offering an innovative solution to overcome tumor microenvironment barriers (38).

In the field of small cell lung cancer (SCLC), research has for the first time elucidated the immune-modulating mechanism of combining PARP inhibitors (including olaparib and talazoparib) with radiotherapy. This combination therapy specifically activates the cGAS-STING signaling pathway and stabilizes the mRNA expression of the chemokine CXCL10 through an EIF4E2-dependent mechanism, thereby promoting T-cell infiltration into the tumor microenvironment and enhancing anti-tumor immune responses (39). This significant finding not only deepens our understanding of the mechanisms underlying PARP inhibitor function but also provides a solid theoretical foundation for combining PARP inhibitors with immune checkpoint inhibitors (such as anti-PD-L1 antibodies). These research outcomes hold promise for bringing new therapeutic breakthroughs to SCLC patients with limited responses to conventional treatments, ushering in a new chapter for personalized precision medicine.

In head and neck squamous cell carcinoma (HNSCC), studies found that the combination of the epidermal growth factor receptor (EGFR) inhibitor cetuximab and the PARP-1 inhibitor olaparib produces significant synergistic radiosensitizing effects. The core mechanism lies in the comprehensive collapse of the DNA damage repair system due to dual-target inhibition—blocking the EGFR signaling pathway impairs cell proliferation and survival, while PARP-1 inhibition disrupts single-strand break repair. Together, they make tumor cells more sensitive to radiation-induced DNA damage, significantly enhancing cell killing (40). Additionally, the novel PARP inhibitor GPI-15427 exhibits a dual mechanism: on one hand, it significantly increases DNA breaks (evidenced by elevated comet tail moment), directly exacerbating genetic damage; on the other hand, it activates the apoptotic pathway, promoting programmed cell death (41). This multi-dimensional mode of action not only improves local control rates of radiotherapy but also provides new directions for developing more potent clinical radiosensitizers.

In the field of oral squamous cell carcinoma (OSCC), research has for the first time revealed that olaparib can significantly enhance the radiosensitivity of tumor cells and effectively suppress their metastatic potential by targeting the IL-17A/NF-κB/p38 signaling axis (42). This finding is particularly important given OSCC’s high propensity for local invasion and lymph node metastasis, and the intervention of this signaling pathway provides a novel molecular target for halting tumor progression. Crucially, in addressing the clinically challenging radioresistant OSCC, studies have confirmed that olaparib can specifically inhibit PARP1 overexpression, successfully reversing the radioresistance of FaDu-RR cells (43). This breakthrough not only suggests PARP1 overexpression as a potential biomarker for radioresistance but also demonstrates that targeting PARP1 is an effective strategy to overcome radiotherapy tolerance in OSCC. These findings offer a feasible new therapeutic approach for patients with refractory OSCC, highlighting the great potential of personalized combination therapy in improving prognosis.

In the treatment of triple-negative breast cancer (TNBC), PARP1 inhibitors exhibit significant radiosensitizing effects. In inflammatory breast cancer (IBC) models, PARP1 inhibitors achieve radiosensitization by delaying DNA double-strand break repair, an effect partially dependent on BRCA1 mutation status (44). Studies have shown that the combination of olaparib and the PI3K inhibitor PI-103 synergistically enhances radiosensitivity: PI-103 induces a “BRCAness” phenotype by downregulating BRCA1 expression, a synergy validated in MDA-MB-435S and MDA-MB-231-BR cell models (45). These findings provide a theoretical foundation for precision radiotherapy in TNBC and other breast cancer patients with DNA repair deficiencies.

In prostate cancer radiosensitization research, the application of functional ex vivo assays enabled, for the first time, the direct observation of PARP1-dependent end joining (PARP1-EJ) repair switching in patient tumor samples. This characteristic was significantly correlated with sensitivity to PARP inhibitor-mediated radiosensitization (observed in approximately 30% of patient samples with tumor-specific expression), providing a novel biomarker for individualized treatment (46). Mechanistic studies indicate that the combination of PARP inhibitors and radiotherapy promotes tumor cell senescence by inducing cumulative DNA damage, triggering increased p21 expression and elevated β-galactosidase activity. Notably, this senescence-dependent radiosensitization is cell-type specific—significantly delaying tumor regrowth in PC-3 xenograft models but showing no pronounced effect in DU-145 models (47). These discoveries provide guidance for precision radiotherapy in prostate cancer: PARP1-EJ repair switching can serve as a criterion for patient selection, while effectively inducing tumor cell senescence is a key mechanism for successful treatment.

In radiosensitization studies across various solid tumors, PARP inhibitors demonstrate diverse mechanisms of efficacy enhancement. In cervical cancer models, folate-conjugated olaparib nanoparticles (ATO) significantly increased local drug concentration within tumors through an active targeting delivery system, achieving a sensitization enhancement ratio (SER) of 3.81, with confirmed correlation to promoted apoptosis (48). For glioblastoma, a highly radioresistant tumor, the combination of the PARP inhibitor E7016 and temozolomide produced a dual DNA damage effect: not only persistently increasing the number of γH2AX foci (P < 0.01) but also significantly extending tumor growth delay by 6 days compared to conventional treatment through the induction of mitotic catastrophe (49). In neuroendocrine tumors, the combination of olaparib and peptide receptor radionuclide therapy (PRRT) exhibited clear tumor type-dependent synergy, observed only in specific molecular subtypes of xenograft models (P < 0.05), underscoring the need for developing corresponding molecular classification standards to guide clinical application (50). These cross-tumor studies collectively reveal the broad application prospects of PARP inhibitors as radiosensitizers and the necessity for their precise application.

In summary, PARP1-targeted radiosensitization strategies have demonstrated significant potential across various solid tumors. Through mechanisms such as inducing synthetic lethality, modulating DNA repair pathways, and activating immune responses, they markedly enhance radiosensitivity (**Table 2**). Preclinical studies confirm that PARP inhibitors can overcome tumor microenvironment barriers and synergize with immunotherapy and novel nanomedicines, providing a robust theoretical foundation for precision radiotherapy and clinical translation.

**Table 2:** Preclinical evidence for PARP1 inhibitor-mediated radiosensitization.

Tumor type	PARP inhibitor(s)	Radiation parameters	PARPi dose/formulation	Key outcomes	Cell lines/species	Ref.
NSCLC	Olaparib	*In vitro*: Colony formation; *In vivo*: 2 Gy/fx × 5 days	*In vitro*: 4.4 μmol/L (IC_10_); *In vivo*: NS	- SER = 1.211; - ↑Apoptosis & DNA damage	Lewis lung carcinoma/Mouse	(35)
NSCLC	Olaparib	*In vitro*: Clonogenic; *In vivo*: 10 Gy (single)	*In vitro*: NS; *In vivo*: 50 mg/kg (3 days)	- Hypoxia-enhanced radiosensitization; - Mechanism: RAD51 downregulation	Calu-6, Calu-3/Mouse	(36)
NSCLC	Ola-NPs	*In vivo*: NS	*In vivo*: Ola-NPs (~32 nm)	- High SER (3.81); - Tumor growth inhibition; - DSB repair blockade	Human NSCLC xenograft/Mouse	(38)
SCLC	Ola, Tala, Veli	*In vitro*: Clonogenic; *In vivo*: 2 Gy/fx × 4 days or 8 Gy single	*In vitro*: Ola (100nM-1µM), Tala (20nM); *In vivo*: Ola (50mg/kg), Tala (0.2mg/kg)	- cGAS-STING activation & ↑CXCL10; - ↑CD8+ T-cell infiltration; - Effective triple-combination	SBC5, H82, H526, etc./Mouse (NSG, B6129F)	(39)
HNSCC	Ola + Cetuximab	*In vitro*: 2 Gy; *In vivo*: 2 Gy/fx	*In vitro*: Ola (1µM), Cmab (50nM); *In vivo*: Ola (25mg/kg, IP), Cmab (1mg/kg, IP)	- Dual-target inhibition (PARP1+EGFR); - Persistent DNA damage & NHEJ inhibition; - ↑Senescence & apoptosis	Detroit562, FaDu/Mouse (Nude)	(40)
HNSCC	GPI-15427	*In vitro*: 2 Gy; *In vivo*: 2 Gy/day × 2 days	*In vitro*: 7 µM (LD_50_); *In vivo*: Oral (10–300 mg/kg)	- ↑DSBs & impaired repair; - Dose-dependent tumor growth inhibition; - ↑Apoptosis	JHU006, JHU012/Mouse (BALB/c nude)	(41)
OSCC	Olaparib	*In vitro*: 0–6 Gy; *In vivo*: 4 Gy/fx, 2x/week	*In vitro*: 12 µM; *In vivo*: 30 mg/kg, IP, 2x/week	- Targeted IL-17A/NF-κB/p38 axis; - ↓Metastasis; - IL-17A high = poor prognosis	OML1, OML1-R, MOC2/Mouse (C57BL/6)	(42)
HNSCC	Olaparib	*In vitro*: 0–10 Gy; *In vivo*: 2 Gy/fx × 4	*In vitro*: 2 µg/mL; *In vivo*: 10 µg/g, IP × 4	- Overcame radioresistance; - ↓PARP1 expression; - G2/M arrest & growth inhibition	FaDu, FaDu-RR/Mouse (Athymic nude)	(43)
TNBC	Ola + PI-103	*In vitro*: 2–8 Gy; *In vivo*: 3 Gy/fx, 3x/week	*In vitro*: Ola (1µM), PI-103 (0.4µM); *In vivo*: Ola (10mg/kg, IP), PI-103 (10mg/kg, IP)	- Induced “BRCAness”; - SER_0.05_ = 1.7-2.1; - ↓BRCA1, persistent DNA damage	MDA-MB-435S, MDA-MB-231-BR/Mouse (Nude)	(45)
Prostate	Olaparib	Ex vivo: 2 Gy	Ex vivo: 1 µM	- ~30% “Responders”; - Functional PARP1-EJ repair switch; - Tumor-specific effect	Patient samples, PC3, DU145/Human, Mouse	(46)
Prostate	Veliparib	*In vitro*: 6 Gy; *In vivo*: 6 Gy	*In vitro*: 10 μmol/L; *In vivo*: NS	- Senescence-dependent efficacy (PC-3 only); - G2-M arrest, ↑p21; - Delayed tumor regrowth	DU-145, PC-3/Mouse	(47)
Cervical	Ola (ATO NPs)	*In vivo*: 10 Gy (single)	NPs: ATO (FA-PCEC); Dose: 50 mg/kg/d, IV, 3 days	- Active targeting enhanced efficacy; - ↑γ-H2AX, ↓Ki-67, ↑Apoptosis; - ↑Survival; No toxicity	HeLa, A549/Mouse (BALB/c nude)	(48)
GBM	E7016	*In vitro*: 2–10 Gy; *In vivo*: 4 Gy (single)	*In vitro*: 3-5 µM (pre-IR 6h); *In vivo*: 40 mg/kg, PO	- DEF = 1.4-1.7; - ↑Mitotic catastrophe; - Triple therapy ↑Growth delay	U251, MiaPaCa2, DU145/Mouse (Nude)	(49)
NETs	Olaparib	PRRT: 30 MBq [^177^Lu]Lu-DOTA-TATE (single IV)	*In vivo*: 50 mg/kg, IP, daily × 14 days (start -2d)	- CA20948: ↑TTP (60d vs 41d); - NCI-H69: No benefit; - Tumor-type dependent efficacy	CA20948, NCI-H69/Mouse (BALB/c nude)	(50)

NS, Not Specified; Ola, Olaparib; Tala, Talazoparib; Veli, Veliparib; Cmab, Cetuximab; NPs, Nanoparticles; ATO, Active Targeted Olaparib; SER, Sensitization Enhancement Ratio; DEF, Dose Enhancement Factor; TTP, Time To Progression; RR, Radioresistant; PARP1-EJ, PARP1-dependent end joining; IP, Intraperitoneal; IV, Intravenous; PO, Per Os (Oral).

## 6 Clinical translation of PARP1 inhibitors for radiosensitization

PARP inhibitors (PARPis) have demonstrated potent radiosensitizing potential in preclinical models, driving their rapid entry into clinical exploration in combination with radiotherapy. Current clinical translation efforts primarily focus on locally advanced or unresectable solid tumors, with core objectives of validating the safety and tolerability of combination regimens, assessing preliminary efficacy, and identifying potential beneficiary populations. Despite overall positive progress, challenges remain, including heterogeneity in efficacy across tumor and molecular subtypes, uncertainty regarding optimal dosing strategies, and toxicity management.

### 6.1 Safety and tolerability: dose exploration and toxicity profile

Safety is the primary consideration in the clinical translation of PARPi combined with radiotherapy. Several phase I/II studies, utilizing dose-escalation designs, have preliminarily established the maximum tolerated dose (MTD) and recommended phase II dose (RP2D) for different PARPis in the context of radiotherapy.

In the phase I segment of the SWOG 1206 trial (NCT01386385) for stage III NSCLC (N = 21), veliparib was evaluated at three dose levels (40, 80, and 120 mg BID) during concurrent chemoradiotherapy (CRT). Dose-limiting toxicities (DLTs) occurred in 2 patients (9.5%): one at 40 mg and one at 80 mg. No DLTs were observed at the 120 mg BID dose level, which was consequently selected as the recommended phase II dose and deemed to have a manageable safety profile (51). In a separate phase I trial (NCT02412371) of patients with unresectable stage III NSCLC (N = 48), veliparib was evaluated in combination with carboplatin/paclitaxel-based chemoradiotherapy followed by consolidation chemotherapy. The maximum tolerated dose (MTD) and recommended phase II dose (RP2D) for veliparib were determined to be 240 mg twice daily during chemoradiotherapy, followed by 120 mg twice daily during consolidation. At the RP2D, the most common all-grade adverse events observed throughout the entire treatment period included nausea (83%), esophagitis (75%), neutropenia (75%), and thrombocytopenia (75%), supporting the tolerability of this extended dosing regimen (52). A phase I study (N = 28 patients with loco-regional or oligometastatic NSCLC) investigating olaparib with mildly hypofractionated radiotherapy (66 Gy/24 fx) established the maximum tolerated dose (MTD) as olaparib 25 mg once daily (QD) in the cisplatin-free cohort. However, the regimen combining low-dose daily cisplatin with olaparib proved intolerable, exceeding the MTD due to hematologic and esophageal dose-limiting toxicities (DLTs) (53). Critically, severe late pulmonary toxicity was observed in 5 patients (18%) across all dose levels, including grade 5 (fatal) events, with exploratory analyses indicating an association with the radiation dose to the lungs (53). This underscores lung V20 and mean lung dose as critical risk factors requiring stringent control in PARPi-radiotherapy combinations.

In a phase I trial (COMIRB #11-1658) for high-risk, locally advanced HNSCC patients with heavy smoking histories (N = 16 enrolled, 15 evaluable for toxicity), the combination of olaparib, cetuximab, and radiotherapy was evaluated. The most common grade 3–4 toxicities were radiation dermatitis (38%) and mucositis (69%). Although the maximum tolerated dose (MTD) was determined to be olaparib 50 mg twice daily (BID), the recommended phase II dose (RP2D) was set at 25 mg BID due to an improved toxicity profile, particularly reduced radiation dermatitis (54). These data collectively demonstrate the “double-edged sword” nature of PARPi-mediated radiosensitization—it can enhance tumor killing but may also amplify radiation damage to normal tissues. Therefore, precise radiotherapy planning (e.g., IMRT/VMAT) and strict dose-volume constraints are crucial for ensuring treatment safety.

Looking forward, the successful clinical translation of this combination strategy will depend on systematic efforts to mitigate these risks.The clinical translation of PARP inhibitor-mediated radiosensitization must carefully navigate its “double-edged sword” effect—enhancing tumor control at the cost of increased normal tissue toxicity (e.g., pulmonary injury, mucositis). To mitigate these risks and improve the therapeutic index, a multi-pronged strategy is essential. First, the adoption of advanced radiotherapy techniques (e.g., IMRT/VMAT) coupled with strict dosimetric constraints for organs-at-risk (e.g., lung V20, mean lung dose) is fundamental to minimize physical radiation exposure. Second, optimizing PARPi dosing schedules—such as exploring pulsed administration around RT fractions rather than continuous dosing—may preserve tumor radiosensitization while allowing normal tissue repair. Third, proactive patient monitoring and aggressive supportive care (e.g., nutritional support, pain management) are crucial for managing acute toxicity and maintaining treatment compliance. Finally, future trial designs should incorporate risk-adapted patient selection, consider novel biomarkers for normal tissue susceptibility, and explore phased dose escalation based on treatment site. By integrating these technological, pharmacological, and supportive measures, the oncology community can better harness the promise of PARPi-radiotherapy combinations while safeguarding patient safety. A comprehensive summary of key clinical trials evaluating PARP inhibitors in combination with radiotherapy is provided in (**Table 3**).

**Table 3:** Summary of clinical trials evaluating PARP inhibitors in combination with radiotherapy.

Ref	Trial	NCT number	Patient population	Biomarker status	PARPi	RT schedule	Key endpoints	Efficacy outcomes	Key toxicities (≥G3)
(51)	I/II	NCT01386385	Stage IIIA/IIIB NSCLC	Not specified	Veliparib	60 Gy 30 fractions	Primary: PFS Secondary: OS, RR, Toxicity	PFS: 9.3 mo vs 9.9 mo 1-yr OS: 89% vs 54%	Lymphopenia, Neutropenia, Esophagitis, Fatigue
(52)	I	NCT02412371	Stage III NSCLC	Not specified	Veliparib	60 Gy 30 fractions	Primary: MTD, RP2D Secondary: PFS, OS, ORR	Median PFS: 19.6 mo Median OS: 32.6 mo ORR: 63% (all), 73% (RP2D)	Neutropenia (47.9%), Leukopenia (31.3%), Lymphopenia (27.1%)
(53)	I	NCT01562210	Stage II/III NSCLC	Not specified	Olaparib	66 Gy 24 fractions	Primary: MTD Secondary: Safety, Tolerability, PD	2-yr Local Control: 84% Median OS: 28 mo Median PFS: 12 mo	Esophageal ulcer/stricture (G3), Thrombocytopenia (G4), Pneumonitis/fibrosis (G4-5, 21%)
(54)	I	COMIRB #11-1658	LA-HNSCC	HPV+/-, EGFR+ (87%)	Olaparib	69.3 Gy 33 fractions, 6.5 weeks, IMRT	Primary: Safety, MTD/RP2D Secondary: PFS, OS, LC, DC	2-yr OS: 72% 2-yr PFS: 63% LC: 72% DC: 79%	Radiation dermatitis (38%), Mucositis (69%) Nausea/Vomiting (G3, 1 DLT)
(57)	I	NCT03109080	TNBC post-NACT	HRD+ (58.3%), gBRCA1/2 mut (29.2%)	Olaparib	50–50.4 Gy Boost to 63 Gy	Primary: Safety, MTD Secondary: OS, EFS	3-yr OS: 83% 3-yr EFS: 65%	Acute: G3 dermatitis (8.3%), G3–4 lymphopenia (45.8%) Late: No ≥G3 toxicity
(55)	I	NCT03109080	TNBC post-NACT	BRCA1/2 tumor mut (29.2%)	Olaparib	50 Gy/25 fx or 50.4 Gy/28 fx	Primary: DLT (reported) Secondary: Late toxicity, Efficacy	1-yr OS: 96%	No treatment-related ≥G3 AEs; 1 pt with persistent G2 AEs (pain, fibrosis, distortion)
(56)	I	NCT03109080	TNBC post-NACT	BRCA1/2 tumor mut (29.2%)	Olaparib	50 Gy/25 fx boost to 63 Gy	Primary: DLT (reported) Secondary: Late toxicity, Efficacy	1-yr OS: 96%	No treatment-related ≥G3 AEs; 1 pt with persistent G3 AEs (pain, fibrosis, distortion)
(58)	Ib	NCT01589419	Stage II-III Rectal Adenocarcinoma	Not specified	Veliparib	50.4 Gy 28 fractions 5.5 weeks	Primary: MTD, RP2D Secondary: Safety, Tolerability, Preliminary efficacy, PK	pCR: 29% (9/31) Downstaging: 71% (22/31)	No G3–4 DLT; G3 diarrhea: 9% Common AEs: Nausea (53%), Diarrhea (50%), Fatigue (50%)
(59)	II	ACTRN12615000407594	Glioblastoma	MGMT unmethylated	Veliparib	60 Gy 30 fractions 6 weeks	Primary: PFS-6m	PFS-6m: 46% (Exp) vs 31% (Ctrl) Median OS: 12.7 mo vs 12.8 mo	Thrombocytopenia (17%), Neutropenia (12%), Seizure (11%)
(60)	I-II	NCT01514201	DIPG	Not required (imaging-based)	Veliparib	54 Gy 30 fractions weeks	Primary: RP2D, OS, PFS, Toxicity, TMZ dose escalation feasibility	1-yr OS: 37.2% 2-yr OS: 5.3%	Lymphopenia (32.8%), Neutropenia (32.7%), Leukopenia (30.8%), Thrombocytopenia (23.1%)
(61)	I	NCT02944396	NSCLC	No specific biomarker required; some with PD-L1 expression (median CPS 5)	Veliparib	No RT	Primary: RP2D, Safety, Tolerability, PK, Preliminary efficacy	Confirmed ORR: 40% Best Overall Response: 64%	Anemia (32%), Neutropenia (24%), Thrombocytopenia (16%), Disease progression (12%)

AEs, Adverse Events; CW, Chest Wall; DLT, Dose-Limiting Toxicity; EFS, Event-Free Survival; gBRCA, germline BRCA; HRD, Homologous Recombination Deficiency; LA-HNSCC, Locally Advanced Head and Neck Squamous Cell Carcinoma; LC, Local Control; DC, Distant Control; MTD, Maximum Tolerated Dose; NACT, Neoadjuvant Chemotherapy; NSCLC, Non-Small Cell Lung Cancer; ORR, Objective Response Rate; OS, Overall Survival; pCR, Pathological Complete Response; PD, Pharmacodynamics; PFS, Progression-Free Survival; PK, Pharmacokinetics; RP2D, Recommended Phase II Dose; RR, Response Rate; TNBC, Triple-Negative Breast Cancer.

### 6.2 Efficacy heterogeneity and diversified combination strategies

Although preliminary safety has been established, the clinical efficacy of PARPi combined with radiotherapy shows significant heterogeneity across tumor types and molecular subtypes, suggesting that a one-size-fits-all strategy is unlikely to succeed.

In the phase I RADIOPARP trial (NCT03109080) involving 24 patients with high-risk, early-stage TNBC, the combination of olaparib and postoperative radiotherapy demonstrated an exceptional safety profile. Olaparib was successfully escalated to the maximum planned dose of 200 mg twice daily without observing any dose-limiting toxicities (55, 56). At the 2-year follow-up, no late grade ≥3 treatment-related toxicities were reported, with the maximum observed toxicity being grade 2 breast pain, fibrosis, and deformity in a single patient (4.2%) (57). Regarding efficacy, the 3-year overall survival (OS) was 83% (95% CI: 70%-100%) and event-free survival (EFS) was 65% (95% CI: 48%-88%) (57). Notably, efficacy was independent of homologous recombination (HR) status, suggesting a potential benefit beyond HR-deficient tumors (57). These results provide strong support for the use of PARPis in the adjuvant/neoadjuvant radiotherapy setting for TNBC. In the neoadjuvant setting for locally advanced rectal cancer, a phase 1b trial (NCT01589419) evaluated veliparib combined with capecitabine and radiotherapy. The study enrolled 32 patients, and the recommended phase 2 dose of veliparib was established at 400 mg twice daily without reaching the maximum tolerated dose. The regimen demonstrated a manageable safety profile, with the most common treatment-emergent adverse events being nausea (53%), diarrhea (50%), and fatigue (50%); grade 3 diarrhea occurred in 9% of patients, and no grade 4 events were reported (58). Promising antitumour activity was observed, with tumor downstaging in 71% (22 of 31) of evaluable patients and a pathological complete response (pCR) rate of 29% (9 of 31) (58).

However, in the randomized phase II VERTU trial (NCT02152982) for patients with newly diagnosed, MGMT-unmethylated glioblastoma (N = 125; 84 in the experimental arm, 41 in the control arm), the addition of veliparib to temozolomide-based chemoradiotherapy did not confer a survival benefit. The 6-month progression-free survival (PFS-6m) was 46% (95% CI: 36%-57%) in the experimental arm versus 31% (95% CI: 18%-46%) in the standard arm. Critically, the median overall survival was nearly identical between the two arms: 12.7 months (95% CI: 11.4-14.5) with veliparib versus 12.8 months (95% CI: 9.5-15.8) with standard therapy, indicating no clinical improvement from the combination (59), Similarly, a Pediatric Brain Tumor Consortium phase I/II trial (NCT01514201) for pediatric diffuse intrinsic pontine glioma (DIPG) evaluated veliparib combined with radiotherapy followed by veliparib and temozolomide. The study enrolled 65 eligible patients and established the recommended phase II dose of veliparib during radiation as 65 mg/m² twice daily. However, a planned interim analysis concluded that the regimen did not show a survival benefit compared with historical controls, and accrual was stopped for futility. The 1-year and 2-year overall survival rates were 37.2% (Standard Error [SE] 7%) and 5.3% (SE 3%), respectively, which did not represent an improvement over existing outcomes (60). This efficacy disparity may stem from differences in intrinsic DNA repair capacity, drug permeability across the blood-brain barrier, and the immune microenvironment, underscoring the necessity of evaluating PARPi sensitization effects within specific tumor contexts.

Initial clinical efforts have established the feasibility of combining PARP inhibitors with immune checkpoint inhibitors (ICIs). A phase I dose-escalation study (NCT02944396) evaluated the quadruple combination of veliparib, nivolumab, and platinum-doublet chemotherapy in patients with advanced/metastatic NSCLC (N = 25). The regimen was well-tolerated, with recommended phase 2 doses established for both carboplatin/paclitaxel (veliparib 120 mg BID) and carboplatin/pemetrexed (veliparib 240 mg BID) backbones. The most common any-grade adverse events were fatigue (56%), nausea (52%), and anemia (48%). Promising preliminary antitumor activity was observed, with a confirmed objective response rate (ORR) of 40% and a best overall response of 64% (61). Building on this foundation, and motivated by the preclinical evidence that PARPi and radiotherapy can synergistically activate the cGAS-STING pathway to enhance anti-tumor immunity (39), the logical next step is the integration of radiotherapy. Thus, the “radiotherapy + PARPi + ICI” triple-modality approach represents a crucial direction for future precision oncology, aiming to maximally synergize innate and adaptive immune activation and convert local treatment into a potent, systemic anti-tumor effect.

To translate this robust preclinical rationale into clinical success, future trials must be mechanistically informed. A critical unresolved question is the optimal sequencing of RT, PARPi, and ICI. Based on the mechanism, we propose a “Prime and Amplify” strategy: concurrent administration of RT and PARPi should be used first to “prime” an immune response by inducing cytosolic DNA, activating the cGAS-STING pathway, and promoting T-cell infiltration. This should be followed by the administration of an ICI to “amplify” the pre-activated T-cell response by reversing T-cell exhaustion. This sequence is hypothesized to be more effective than administering the ICI first or giving all three modalities concurrently.

### 6.3 Biomarkers: from exploration to guiding precision therapy

Given the heterogeneity in treatment efficacy, reliable biomarkers are urgently needed to identify patient populations most likely to benefit from PARPi-based combination therapies. Current research has identified several promising candidates: in NSCLC, a phase III trial of veliparib showed that LP52-positive patients had significantly improved overall survival, suggesting its predictive potential (62, 63); in ED-SCLC, patients with high SLFN11 expression derived greater PFS benefit from veliparib combined with chemotherapy (64). These findings reflect a shift from “all-comer” approaches toward precision “enriched-population” strategies. Additionally, functional assays such as the PARP1-EJ repair switch test in prostate cancer offer a direct way to assess tumor sensitivity to PARPis, enabling more individualized treatment (46). Looking forward, a multidimensional biomarker system integrating genomic (e.g., HRD status), transcriptomic (e.g., SLFN11), and functional (e.g., PARP1-EJ) features will be essential to guide the clinical application of PARPi-mediated radiosensitization. Among current candidates, HRD scores and tBRCA mutations are the most clinically validated and readily implementable, while SLFN11 requires further technical standardization, and the PARP1-EJ assay remains in earlier stages of technical and clinical validation.

#### 6.3.1 HRD and BRCA biomarkers

These two biomarkers not only possess the most robust evidence base, but their clinical value has been consistently validated across multiple pivotal Phase III trials. In these registrational studies, the use of PARP inhibitors in biomarker-selected populations consistently demonstrated statistically significant and clinically meaningful improvements in progression-free survival (e.g., hazard ratios [HRs] ranging from approximately 0.2 to 0.7, all with p-values < 0.01). This body of evidence spans ovarian cancer [PAOLA-1 (65), PRIMA (66), SOLO1 (67)], prostate cancer [PROfound (68), TALAPRO-2 (69)], and pancreatic cancer [POLO (70)], with the most extensive validation in high-grade serous ovarian cancer (HGSOC). Mechanistically, their core value lies in directly detecting the fundamental impairment of homologous recombination repair (HRR) function, which represents the therapeutic target of PARP inhibitors (PARPi). This biological rationale has successfully translated into clinical recognition, as evidenced by regulatory approvals of companion diagnostic assays (such as MyChoice CDx and FoundationOne CDx) and the incorporation of these biomarker testing into treatment recommendations by authoritative guidelines including the National Comprehensive Cancer Network (NCCN), thereby firmly establishing their critical role in precisely identifying patients who are most likely to benefit from PARPi treatment.

To advance the broader clinical application of HRD scores and tBRCA mutations, their clinical validation pathway should systematically focus on three core components: First, in terms of detection methodology, there is an urgent need to achieve standardization and normalization of HRD testing through cross-platform concordance studies (such as comparing MyChoice CDx and FoundationOne CDx), while establishing analytical performance standards for tBRCA detection in scenarios like liquid biopsy. Second, for threshold determination, meta-analyses of individual patient data from completed Phase III trials should be conducted to explore the continuous relationship between HRD scores and clinical benefits, thereby validating or optimizing existing interpretive criteria, with confirmation through prospective biomarker-driven studies. Finally, in clinical trial integration, enrichment designs can be adopted for cancer types with established benefits, or biomarker-stratified designs for emerging fields, to accurately assess their predictive value and actively explore their guiding role in combination therapies with immunotherapies and targeted agents.

#### 6.3.2 SLFN11

SLFN11 represents a highly promising biomarker for clinical application, demonstrating unique value in predicting the efficacy of PARP inhibitors. This is most strongly supported by evidence in small cell lung cancer (SCLC). Multiple studies have confirmed a significant positive correlation between SLFN11 expression levels and sensitivity to PARP inhibitors. In patient-derived xenograft (PDX) models, immunohistochemistry-based detection of SLFN11 protein has been validated as a reliable predictive tool (71). Further compelling evidence comes from a randomized, double-blind phase II trial (NCT01638546) in patients with recurrent SCLC (N = 104). While the study did not meet its primary endpoint for the overall population, a pre-planned biomarker analysis revealed that SLFN11-positive patients derived substantial benefit from veliparib combined with temozolomide (TMZ). In this subgroup, treatment with TMZ/veliparib significantly prolonged both median progression-free survival (5.7 months vs. 3.6 months; P = .009) and median overall survival (12.2 months vs. 7.5 months; P = .014) compared to TMZ/placebo (72). This aligns with the trend observed in a first-line ED-SCLC study (64), solidifying SLFN11’s role as a predictive biomarker.

At the mechanistic level, SLFN11 exhibits a clear synergistic effect with PARP inhibitors: when PARP inhibitors cause replication fork stalling through “PARP trapping,” SLFN11 is specifically recruited to DNA damage sites, where it disrupts the stability of RPA-ssDNA complexes, inhibits the homologous recombination repair pathway and DNA damage checkpoint maintenance, ultimately leading to cell death (73, 74). These systematic clinical studies and mechanistic investigations collectively establish SLFN11 as a novel biomarker independent of BRCA status and suggest its potential to significantly expand the population of patients who may benefit from PARP inhibitors.

To advance the clinical translation of SLFN11, we recommend using immunohistochemistry for SLFN11 detection and determining the optimal cutoff through receiver operating characteristic curve analysis based on clinical outcomes in larger validation cohorts. In terms of clinical trial design, phase III confirmatory studies incorporating SLFN11 biomarker stratification should be conducted in SCLC, with SLFN11-positive patients serving as the enriched population to systematically evaluate the efficacy and safety of PARP inhibitor combination regimens in first-line treatment, thereby providing definitive evidence for their clinical application.

#### 6.3.3 LP52

LP52 is a candidate biomarker in the advanced translational research phase that has undergone exploratory prospective clinical validation. Its key supporting evidence comes from a Phase III randomized trial (NCT02264990) in first-line advanced non-squamous NSCLC. The study prospectively utilized a clinically validated HTG assay for LP52 stratification. In the LP52-positive subgroup (n=80, 13% of the total population of N = 595), patients receiving veliparib combined with carboplatin/paclitaxel showed a median overall survival of 11.2 months versus 9.2 months with chemotherapy alone (hazard ratio [HR] 0.644, 95% confidence interval [CI]: 0.396-1.048). However, the primary endpoint was not met (P = .113 for the OS difference in the LP52+ subgroup), and no OS benefit was observed in the overall population (HR 0.986, 95% CI: 0.827-1.176; P = .846) (75). The trend observed in the LP52+ subgroup was limited by the small sample size of the biomarker-defined population, indicating its potential while highlighting the need for validation in a larger, enriched cohort.

To accelerate the clinical translation of LP52, it is recommended to prioritize the standardization of the detection system and establish the optimal cutoff value through large-scale retrospective cohort studies based on clinical endpoints. On this basis, confirmatory clinical trials should be conducted using a prospective biomarker-stratified design. Specifically, an enrichment study design could be implemented in non-small cell lung cancer, where LP52-positive patients would serve as the core study population and be randomized to either PARP inhibitor combination therapy or standard treatment groups. This approach would systematically validate its predictive efficacy and promote clinical application.

#### 6.3.4 PARP1-EJ

PARP1-EJ functional assay represents a mechanistically grounded yet clinically underdeveloped biomarker. Its principle lies in directly assessing tumor cells’ reliance on the PARP1-EJ pathway - an alternative repair mechanism - when confronting DNA damage induced by radiotherapy combined with PARP inhibition. The preclinical validation of this concept stems from sequential investigations: initial work first demonstrated in tumor cell lines that the “switch” to PARP1-EJ repair constitutes the key mechanism underlying olaparib-mediated radiosensitization, a process independent of homologous recombination status (64). Subsequent research developed an ex vivo functional assay capable of detecting this repair switch in fresh prostate cancer tissue slices, observing this phenomenon in approximately 30% of patient samples, thereby suggesting its potential clinical applicability (46). Nevertheless, this assay faces significant challenges: its methodology depends on ex vivo culture of fresh tumor tissue and high-content imaging analysis, creating complex procedures difficult to standardize across multiple centers. Furthermore, the cutoff values (such as the PARP enhancement ratio, PIER) for distinguishing “responders” require clearer definition through prospective studies. Consequently, while PARP1-EJ offers a unique functional perspective for prediction, it currently serves better as a stratification or enrichment biomarker in exploratory clinical trials rather than a tool ready for guiding routine clinical practice.

In summary, the clinical translation of PARP inhibitor combined with radiotherapy has evolved along a comprehensive pathway encompassing safety validation, efficacy exploration, and precision stratification. Current research has not only established safety profiles and toxicity characteristics across different tumor types but has also revealed the complex mechanisms underlying efficacy heterogeneity. More importantly, a multi-level biomarker system has been developed, ranging from traditional HRD/tBRCA to emerging markers such as SLFN11, LP52, and the PARP1-EJ functional assay. Future research should focus on: optimizing normal tissue toxicity control through more precise radiotherapy techniques and individualized dosing; developing accurate patient stratification strategies based on multidimensional biomarkers; and actively exploring innovative combination modalities, particularly with immunotherapy. Ultimately, by integrating biomarker-guided precision medicine concepts into clinical trial design, we can maximize the radiosensitizing efficacy of PARP inhibitors, advancing this strategy from research exploration to clinical practice and providing more patients with individualized treatment options.

## 7 Resistance mechanisms and rational combination strategies in radiosensitization

The evolution of therapy resistance is a hallmark of cancer and a central obstacle in oncology. The combination of PARP inhibitors with radiotherapy is no exception to this rule. Understanding and overcoming the diverse resistance mechanisms is therefore a prerequisite for maximizing the durability and scope of clinical benefit from this strategy (76).

Despite the promising application prospects of PARP inhibitors as radiosensitizers, the emergence of drug resistance remains a major challenge in their clinical implementation. In the context of radiosensitization, the primary resistance mechanisms include: restoration of homologous recombination function (77, 78) (e.g., through secondary mutations in BRCA1/2 genes), mutations in the PARP1 gene (79, 80) (which impair PARP1 trapping on DNA), and enhanced replication fork protection (81, 82) (preventing the collapse of replication forks into double-strand breaks). To overcome these resistance mechanisms, rationally optimized combination strategies are required.

### 7.1 Combination therapy targeting PARP1 and ATR/CHK1 pathways

HGSOC is frequently characterized by TP53 mutations and homologous recombination (HR) deficiency, providing a theoretical basis for the application of PARP inhibitors (PARPis) (83). Research indicates that combined inhibition of PARP1 and the ATR/CHK1 pathway is an effective strategy to overcome this resistance.

The ATR/CHK1 pathway regulates the DNA damage response, cell cycle checkpoints, and replication fork stability. In PARPi-resistant cells with restored HR function, an “acquired vulnerability” exists, manifesting as a strong dependence on ATR-mediated RAD51 focus formation and replication fork protection. Inhibition of ATR/CHK1 can synergize with PARPis through the following mechanisms: abrogating S-phase and G2/M cell cycle checkpoints, forcing cells with unrepaired DNA damage into mitosis and triggering mitotic catastrophe; and interfering with replication fork protection, leading to the collapse of stabilized replication forks (84).

Preclinical studies have confirmed that ATR inhibitors (e.g., VE-821, ceralasertib/AZD6738) can restore sensitivity to PARPis in BRCA1-mutated resistant cells and demonstrate synergistic antitumor activity in HGSOC patient-derived xenograft (PDX) models, even inducing complete tumor regression (85, 86). Furthermore, the combination of CHK1 inhibitors (e.g., prexasertib, MK-8776) with olaparib can reestablish PARPi sensitivity in HGSOC models with BRCA reversion mutations. Preliminary Phase I clinical trials suggest that this combination regimen provides clinical benefit for BRCA1-mutated HGSOC patients who have progressed on prior PARPi therapy (87).

In summary, these collective findings indicate that PARPi-resistant cells rely more heavily on the functional integrity of the ATR/CHK1 pathway than sensitive cells. Therefore, the combination of PARP inhibitors with ATR or CHK1 inhibitors represents a promising precision therapy strategy capable of reversing and overcoming multiple PARPi resistance mechanisms.

### 7.2 Combined immune checkpoint therapy

Immunotherapy targeting immune checkpoint pathways has made significant contributions to the treatment advancement of various tumors. Immune checkpoint therapy is based on novel drugs that enhance anti-tumor immunity and mitigate the immunosuppressive microenvironment through checkpoint molecules, which are surface receptors on immune cells that regulate immune responses.

Numerous ongoing clinical trials are currently investigating the potential of combining immune checkpoint inhibitors (ICI) with various therapeutic approaches for cancer treatment, with the combination strategy involving PARP inhibitors (PARPi) attracting particular attention. For instance, the phase I/II TOPACIO/Keynote-162 trial (NCT02657889) evaluated niraparib combined with pembrolizumab in a pooled cohort of patients with recurrent ovarian cancer (N = 62; 60 evaluable for efficacy). The combination demonstrated promising activity in this heavily pre-treated population, with an objective response rate (ORR) of 18% (90% CI: 11%-29%) and a disease control rate of 65% (90% CI: 54%-75%). Notably, these responses were observed regardless of platinum sensitivity, BRCA mutation status, or homologous recombination deficiency (HRD) status (88).Notably, however, in the ovarian cancer cohort of another study (NCT02484404), 77% of enrolled patients were BRCA wild-type (BRCAwt) and showed poorer response to olaparib combined with durvalumab. In contrast, a single-center, proof-of-concept phase II trial (NCT02484404) of olaparib combined with durvalumab in heavily pre-treated, recurrent ovarian cancer (N = 35; median of 4 prior lines, 86% platinum-resistant, 77% BRCA wild-type) demonstrated more modest clinical activity. The objective response rate (ORR) was 14% (5/35; 95% CI: 4.8%-30.3%), with a median progression-free survival (PFS) of 3.9 months (89). Despite the limited clinical efficacy, paired biomarker analyses revealed that treatment induced a significant immunostimulatory environment, characterized by enhanced IFNγ and CXCL9/CXCL10 expression. Importantly, increased IFNγ production was associated with significantly improved PFS (HR, 0.37; 95% CI: 0.16-0.87; P = 0.023), while elevated VEGFR3 levels were associated with worse PFS (HR, 3.22; 95% CI: 1.23-8.40; P = 0.017), suggesting potential mechanisms of response and resistance (89).

Despite these variable outcomes, the combination of PARP inhibitors with immune checkpoint blockade remains a highly promising direction in cancer therapeutics. The rationale for this approach is grounded in the ability of PARPi to enhance response to ICI treatment and upregulate anti-tumor immune activity. The scientific community continues to closely monitor emerging data on the efficacy of ICI in ovarian cancer. While current understanding of the effectiveness of various ICI-based combination therapies in PARPi-resistant cancer patients remains limited, several preliminary reports have indicated the potential of PARPi-ICI combinations to prevent the development of PARPi resistance (90).

Among current investigational strategies, the combination with ATR inhibitors stands out as particularly compelling due to its robust mechanistic rationale for targeting multiple resistance pathways—including fork protection and cell cycle checkpoints—coupled with the clinical feasibility of managing its predictable yet significant toxicity profile. Initial clinical trials should prioritize evaluating this combination in selected patient populations with aggressive, radioresistant malignancies.

## 8 Summary and future perspectives

PARP1 plays a central role in the DNA damage response induced by radiotherapy in tumors. Through mechanisms such as recognizing DNA breaks, catalyzing PARylation, and regulating base excision repair (BER), it significantly influences radiotherapy efficacy and cellular fate. Studies have demonstrated that PARP inhibitors (PARPis) can markedly enhance tumor cell sensitivity to radiation by inhibiting DNA repair, inducing synthetic lethality, and modulating the immune microenvironment. Preclinical studies have confirmed the radiosensitizing effects of PARPis across various solid tumors—including non-small cell lung cancer, head and neck cancer, and triple-negative breast cancer—and have further optimized efficacy through strategies such as nanodelivery and combination targeted therapy. In clinical translation, multiple phase I/II trials have validated the safety of combining PARPis with radiotherapy and preliminarily demonstrated survival benefits in certain patient subgroups. However, efficacy remains heterogeneous across tumor types and molecular subtypes, and challenges such as optimal combination regimens, dose optimization, and biomarker identification remain unresolved. Furthermore, the mechanisms of PARPi resistance, long-term toxicity management, and synergistic strategies with other treatments—such as immune checkpoint inhibitors—require further investigation.

Future translation of PARP inhibitor-mediated radiosensitization should be guided by the following research priorities, as evidenced by the body of this review:

Advancing Biomarker-Guided Precision Therapy: Future efforts must focus on validating and implementing a multi-dimensional biomarker system to accurately identify patients who will benefit. This includes not only the clinically established HRD scores and tBRCA mutations, but also emerging biomarkers such as SLFN11 expression and functional assays like the PARP1-EJ repair switch, to move beyond a “one-size-fits-all” approach.

Optimizing Rational Combination Strategies: Preclinical and early clinical data strongly support the exploration of multimodal combinations. A key priority is the clinical validation of the “RT + PARPi + ICI” triple-combination, leveraging the proven ability of PARPis and RT to activate the cGAS-STING pathway and enhance T-cell infiltration. Furthermore, combinations with ATR/CHK1 inhibitors represent a promising strategy to overcome acquired resistance mechanisms, including replication fork protection and HR restoration.

Conquering Resistance Mechanisms: A critical hurdle is the inevitable emergence of resistance. Research must decipher the molecular underpinnings, such as BRCA reversion mutations and PARP1 gene mutations, and advance mechanism-driven reversal strategies. The combination of PARPis with ATR inhibitors has shown preclinical efficacy in resensitizing resistant models by targeting backup DNA repair pathways and disrupting fork protection, marking a vital direction for clinical development (**Table 4**).

**Table 4:** Strategic framework for future development of PARP inhibitor radiosensitizers.

Priority area	Key objectives & strategies	Key evidence & targets	Priority area
Precision Patient Stratification	Develop and validate multi-dimensional biomarker systems beyond BRCA/HRD for trial enrichment and clinical guidance.	Core Biomarkers: HRD score, tBRCA mutations Emerging Biomarkers: SLFN11 expression, LP52, PARP1-EJ functional repair switch	Precision Patient Stratification
Rational Combination Therapy	Explore multimodal regimens to overcome resistance, broaden efficacy, and stimulate systemic immunity. Focus on validating triple-combination and targeted partnerships.	Core Strategy: “RT + PARPi + Immune Checkpoint Inhibitor” Key Targets: cGAS-STING pathway, ATR/CHK1 inhibitors	Rational Combination Therapy
Resistance Mechanism Reversal	Decipher molecular mechanisms of acquired resistance and develop/validate effective combination strategies to reverse it.	Core Mechanisms: BRCA reversion mutations, PARP1 mutations, Replication fork protection; Reversal Strategy: Combine with ATR inhibitors to target backup repair pathways	Resistance Mechanism Reversal

Finally, the successful translation of PARP inhibitor-based radiosensitization from a compelling biological concept to a mainstream clinical modality hinges on fostering proactive and deep interdisciplinary collaboration. The complexities inherent in optimizing this strategy—from understanding the nuances of DNA repair biology and PARP inhibitor pharmacology to delivering highly precise radiotherapy and harnessing the resulting anti-tumor immune response—transcend the boundaries of any single discipline. Future progress will require the concerted efforts of radiation oncologists, molecular biologists, immunologists, medical physicists, pharmacologists, and computational biologists working in integrated teams. Such collaboration is essential to design smarter clinical trials that are underpinned by robust mechanistic hypotheses, to develop and validate multidimensional biomarker platforms for patient selection, to engineer novel drug delivery systems that overcome physiological barriers, and to leverage artificial intelligence for analyzing complex datasets to uncover novel predictors of response and toxicity. By embracing this collaborative paradigm, the field can accelerate the development of safe, effective, and personalized combination therapies that fully realize the potential of PARP inhibitors in improving cancer outcomes.

## Supplementary Material

Supplementary Table 1 Appendix: glossary of abbreviations.
