# Supplementary material for: PARP inhibitors as radiosensitizers: a comprehensive review of preclinical evidence and clinical applications
Source: Front Oncol. 2025 Nov 24;15:1702121. doi: 10.3389/fonc.2025.1702121 (PMC12682679; doi:10.3389/fonc.2025.1702121)
Supplement: Supplementary Table 1 — Appendix: glossary of abbreviations. [file Table1.docx]

| **Abbreviation** | **Full Name** | **Abbreviation** | **Full Name** |
| --- | --- | --- | --- |
| ATR | Ataxia Telangiectasia and Rad3-Related | DIPG | Diffuse Intrinsic Pontine Glioma |
| BER | Base Excision Repair | DLT | Dose-Limiting Toxicity |
| BRCA | BReast CAncer gene | DSB | Double-Strand Break |
| CAT | CATalytic domain (of PARP1) | EFS | Event-Free Survival |
| cGAS-STING | cyclic GMP-AMP Synthase-Stimulator of Interferon Genes | EGFR | Epidermal Growth Factor Receptor |
| CHK1 | Checkpoint Kinase 1 | FA | Folic Acid |
| CRT | ChemoRadiation Therapy | GBM | Glioblastoma |
| DBD | DNA-Binding Domain | HGSOC | High-Grade Serous Ovarian Cancer |
| DDR | DNA Damage Response | HNSCC | Head and Neck Squamous Cell Carcinoma |
| HR | Homologous Recombination | HRD | Homologous Recombination Deficiency |
| ICI | Immune Checkpoint Inhibitor | IMRT | Intensity-Modulated Radiation Therapy |
| LP52 | (A specific biomarker signature) | MGMT | O^6^-Methylguanine-DNA Methyltransferase |
| MTD | Maximum Tolerated Dose | NAD^+^ | Nicotinamide Adenine Dinucleotide |
| NHEJ | Non-Homologous End Joining | NLS | Nuclear Localization Signal |
| NSCLC | Non-Small Cell Lung Cancer | ORR | Objective Response Rate |
| OS | Overall Survival | PAR | Poly(ADP-ribose) |
| PARP1 | Poly (ADP-ribose) Polymerase 1 | PARP1-EJ | PARP1-dependent End Joining |
| PARPi | PARP inhibitor | pCR | Pathological Complete Response |
| PD | Pharmacodynamics | PDX | Patient-Derived Xenograft |
| PFS | Progression-Free Survival | PIER | PARP Inhibition Enhancement Ratio |
| PK | Pharmacokinetics | PRRT | Peptide Receptor Radionuclide Therapy |
| RP2D | Recommended Phase II Dose | RT | Radiation Therapy |
| SER | Sensitization Enhancement Ratio | SLFN11 | Schlafen Family Member 11 |
| SSB | Single-Strand Break | tBRCA | tumor BRCA |
| TNBC | Triple-Negative Breast Cancer | TMZ | Temozolomide |
| VMAT | Volumetric Modulated Arc Therapy | WGR | Trp-Gly-Arg domain (in PARP1) |
| XRCC1 | X-ray Repair Cross-Complementing protein 1 |  |  |
